# Analysis of contributory factors to incidents related to medication assistance for residents taking medicines in residential care homes for the elderly: a qualitative interview survey with care home staff

**DOI:** 10.1186/s12877-022-03016-4

**Published:** 2022-04-22

**Authors:** Hayato Kizaki, Daisuke Yamamoto, Hiroki Satoh, Kotaro Masuko, Hideyuki Maki, Yukari Konishi, Satoko Hori, Yasufumi Sawada

**Affiliations:** 1 Division of Drug Informatics, Keio University Faculty of Pharmacy, 1-5-30 Shibakouen, Minato-ku, Tokyo, 105-0011 Japan; 2 SOMPO Care Inc, 4-12-8 Higashishinagawa, Shinagawa-ku, Tokyo, 140-0002 Japan; 3 Graduate School of Pharmaceutical Sciences, The University of Tokyo, 7-3-1 Hongo, Bunkyo-ku, Tokyo, 113-0033 Japan

## Abstract

**Background:**

In Japan, staff who are not doctors or nurses can assist the elderly in residential care facilities to take their pre-packaged medicines. Therefore, there is a potential risk of incidents specific to staffs. The aim of this study was to clarify the causes of incidents related to medication assistance by staff in residential care facilities.

**Method:**

Semi-structured interviews with staff involved in medication incidents in long-term care facilities, focusing on how and why each incident happened, were conducted. The interview covered basic information about the subject and resident, the circumstances under which the incident had occurred, contributing factors, and countermeasures put in place. Verbatim transcripts of the interviews were prepared. Based on thematic analysis, codes and themes were created.

**Results:**

Twelve subjects participated in this study. All subjects were staffs (not doctors or nurses) in long-term care facilities. All incidents covered in this study were incidents in which the wrong resident was given the medication. The incidents arose because of “not following procedures”, such as lack of “self-check of residents’ faces/residents’ names/residents’ medicine envelopes” or “double-check with other staff” or “using a device for medication intake”. Contributory factors were grouped into four categories: individual resident factor items such as “decreased ability to understand their medication” or “refusal to take medicines”, individual staff factor items such as “lack of knowledge related to medication” or “mental burden” or “experience in medication assistance”, team factor items such as “failure to communicate with other staff”, work environment factor items such as “presence of other residents” or “other work besides medication assistance” or “not enough time” or “little understanding of fostering a safety culture at the facility”.

**Conclusion:**

This study identified four categories of contributory factors that may lead to incidents during medication assistance by caregivers for residents of care homes. These findings should be helpful for risk management in residential care facilities where staff usually provide medication assistance. Separation of meal times and medication assistance, and professional review to stagger the timing of administration of residents’ medication may be effective in reducing incidents.

**Supplementary Information:**

The online version contains supplementary material available at 10.1186/s12877-022-03016-4.

## Introduction

Aging population is a common problem not only in developed countries, but also in developing countries [1]. This is a particular problem in Japan, where the old-age dependency ratio (OADR; persons aged 65 or older per 100 persons aged 20–64 years) was 51 in 2019, the highest in the world [2]. It is predicted that the OADR of more than eight countries will be higher than 70 by 2050, led by Japan, where the OADR is expected to reach 81 [2]. Thus, care of the elderly is becoming a common challenge all over the world.

In Japan, there are many types of facilities for the elderly, including special nursing homes, geriatric health services facilities, nursing care homes, and residential homes with long-term care services. These are not medical facilities, and they do not offer advanced medical treatment. However, nurses carry out routine treatments, such as care of pressure ulcers, suction, breathing assistance, or checking vital signs. Moreover, regular staff, who are not doctors or nurses, often help residents to take medicines that have been pre-packaged. However, it has been reported that medication administration errors occurred more frequently in long-term care homes than in patients’ homes [3]. Furthermore, many residents in long-term care homes take multiple drugs, and medication errors or incidents that might cause adverse effects may occur. The factors contributing to medication errors or incidents during medication administration in hospital or community care settings have been investigated [4–6]. In the case of medication incidents involving nurses, it was found that carelessness, omission, lack of knowledge, lack of guidelines, inappropriate communication, and high workload were contributory factors [7]. On the other hand, the factors contributing to incidents during medication assistance by regular staff have not been investigated.

In the present study, a qualitative interview survey focused on factors contributing to incidents in medication assistance by staff in residential care facilities was conducted. The purpose of this study was to identify the contributing factors in order to improve risk management in residential care facilities.

## Methods

### Design

This study was a multicentre prospective qualitative study. Individual semi-structured interviews were conducted because this method is well suited for the purpose of investigating peoples’ experiences of a focused phenomenon. The semi-structured method also makes it easy for participants to share their experiences. The interviews were conducted with staff who had experienced medication incidents related to assistance of residents in taking their medicines. “Medication incidents” were defined as events in which appropriate medication was not given (including omission of medication) or inappropriate medication was about to be given as a result of medication assistance by staff. The interviews were transcribed verbatim, then coding and themes were generated using thematic analysis, and a conceptual diagram was created.

### Participants

Staffs in long-term care facilities who had experienced medication incidents as defined above were recruited as candidates for interview. All facilities were in the Kanto district (the megalopolis of Tokyo and its suburbs) in Japan.

### Data collection

Semi-structured, face-to-face interviews were conducted according to the interview guide (appendix 1) and took around 1 h. Before the interview, each participant was provided with an explanation about the purpose and method of the research, ethical considerations, and protection of privacy, and their informed consent was obtained. For the interview, the incident report and basic information about residents, such as prescriptions, were prepared in anonymized form.

The interview took place in a private room in the facility where the interviewee worked. All the facilities were in Tokyo, Kanagawa, Saitama, or Chiba in Japan. In the interview, each participant gave basic information about themselves and the resident, details of the medication incident, and their opinion about contributory factors. All interviews were conducted in Japanese. Interviewers were KH, then a Ph.D. student at the Graduate School of Pharmaceutical Sciences, The University of Tokyo, and YD, an employee of SOMPO Care Inc., who provided business support. SH, an associate professor at the Graduate School of Pharmaceutical Sciences, The University of Tokyo, attended some of the interviews. The participants were unknown to KH, YD, and SH. Interviews were audio-recorded with participants’ consent. The authors confirm that all participant/resident identifiers have been removed so that anonymity is assured.

### Data analysis

Verbatim transcripts of the interviews were prepared and thematic analysis was conducted. The analysis theme was “HOW and WHY the medication incident happened”. KH performed all coding inductively. The software used for this analysis was MAXQDA 2020 (VERBI GmbH). Coding units were defined as a set of semantic units. Inductive coding was repeated and codes were modified as appropriate in the process of the analysis. Based on the obtained codes, the theme was created. In this study, in order to enhance the trustworthiness of data analysis, the codes and themes generated by the first author (KH) were reviewed by another author (SH), and then modified and finalized by discussion between KH and SH. Data analysis was conducted at the Laboratory of Drug Lifetime Management in the University of Tokyo Faculty of Pharmaceutical Sciences and the Laboratory of Drug Informatics in the Keio University Faculty of Pharmacy.

### Ethics

All methods were carried out in accordance with the Declaration of Helsinki. This study was approved by the Research Ethics Review Committee, Keio University Faculty of Pharmacy (accession number 190119–1) and by the Research Ethics Review Committee of the Faculty of Pharmaceutical Sciences, the University of Tokyo (accession number 29–14, approved on November 8, 2017).

## Results

### Background of the interviewees

Twelve staff participated in this study. Four were in the 20’s, one was in the 30’s, and 7 were in the 40’s. Seven were males and 5 were females (Table 1).

**Table 1:** Background of the interviewees

	Gender	age	Years of long-term care experience
A	Female	20’s	3—5 years
B	Female	40’s	5 years or more
C	Male	30’s	Less than 1 year
D	Male	20’s	1 – 3 years
E	Male	20’s	5 years or more
F	Male	40’s	5 years or more
G	Female	20’s	5 years or more
H	Female	40’s	1 – 3 years
I	Male	40’s	5 years or more
J	Male	40’s	1 – 3 years
K	Male	40’s	1 – 3 years
L	Female	40’s	Less than 1 year

### Not following procedures

**Fig. 1:**
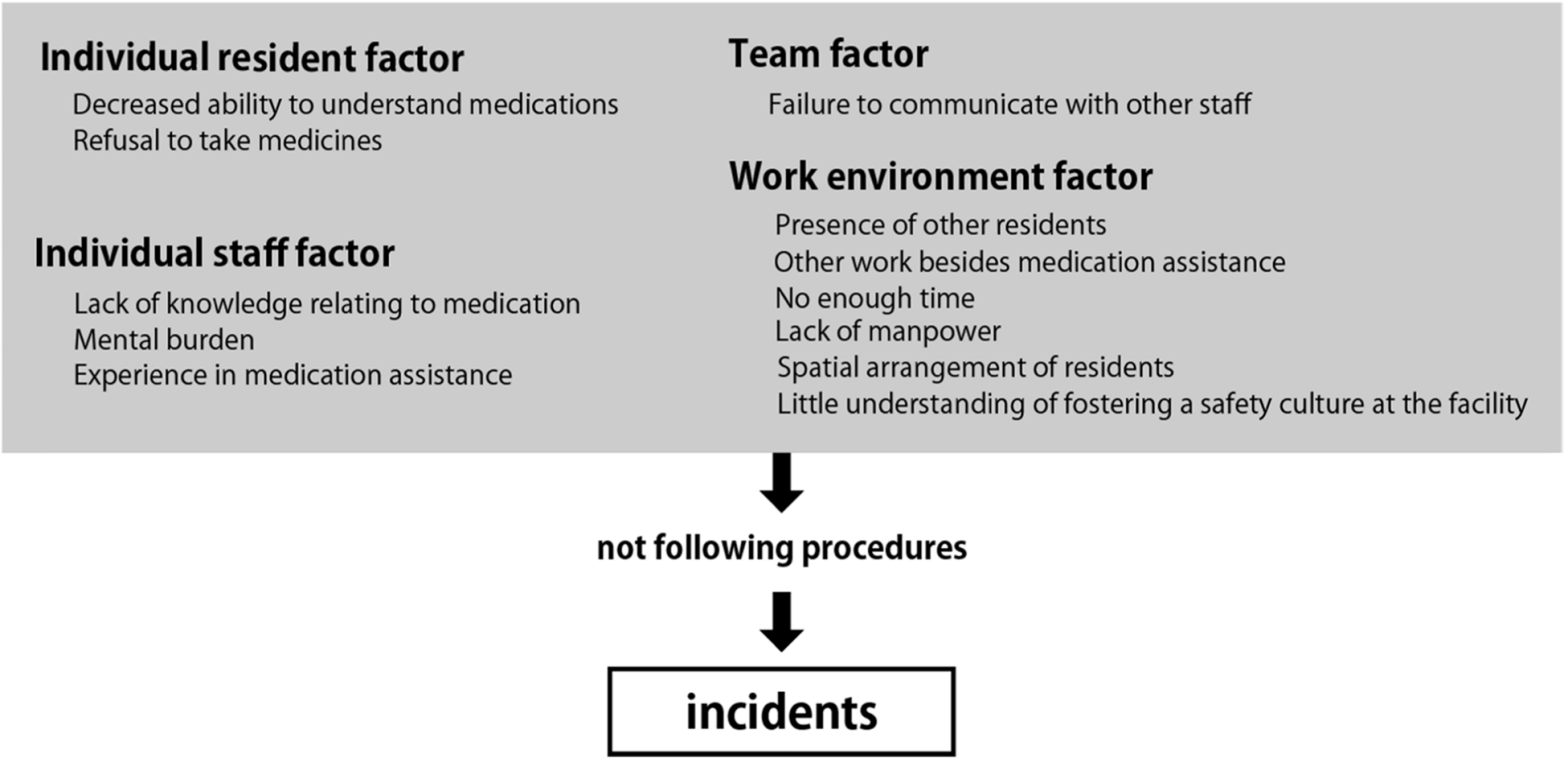
Process of incidents

All incidents in this study involved the wrong resident being given the medication, but none had a serious outcome. Common procedures for medication assistance were in place where the participants worked (appendix 2). The created codes and themes are shown in Table2. The incidents arose because staff omitted some procedures, such as “self-check of residents’ faces/residents’ names/residents’ medicine envelopes” or “double-check with other staff” or “using a device for medication intake”. The contributing factors were classified into 4 categories; individual resident, individual staff, team, work environment. The relationship between each theme and the occurrence of incidents is shown in Fig. 1.

**Table 2:** Codes and themes

Theme	Code
**Not following procedures**	self-check of residents’ faces/residents’ names/residents’ medicine envelopes
	double-check with other staff
	using a device for medication intake
**Individual resident factor**	
Decreased ability to understand medications	recognizing others’ drugs as their own drugs
	a decline of the residents’ understanding about their medication
Refusal to take medicines	refusal to take their medicines
**Individual staff factor**	
Lack of knowledge related to medication	not knowing the resident’s medication
	can’t understand the information on medicines even if they access it
	not afraid of not knowing about the medication
Mental burden	impatience
	assumptions
	decline in concentration
Experience in medication assistance	unfamiliarity with medication assistance
	familiarity with medication assistance
	medication assistance after vacation
**Team factor**	
Failure to communicate with other staff	expectations of support from other staff
	lack of communication
**Work environment factor**	
Presence of other residents	worrying about other residents
	similarity with other resident’s face or name
Other work besides medication assistance	presence of other tasks
No enough time to spare	heavy workload
	delay in tasks
Lack of manpower	burden of delivering medication assistance alone
	lack of manpower
Spatial arrangement of residents	spatial arrangement of residents
Little understanding of fostering a safety culture at the facility	an environment that makes it difficult for staff to follow the manual
	awareness of avoiding making mistakes
	sharing of accident details in the facility
	the occurrence of near-misses

### Individual resident factor

In general, medication assistance is not unilateral, but involves an interaction between the resident and the staff member. “Decreased ability to understand their medication” was a factor in some individual residents. Some residents had “a decline of the residents’ understanding about their medication”, and sometimes incorrectly “recognized others’ drugs as their own”. Because some residents could not recognize their own medication, they would not be able to notice that the wrong medicine was being provided even if it is pre-packaged. Moreover, residents who showed “refusal to take their medicines” may not have understood what they were taking and therefore might not be able to recognize errors.

### Individual staff factor

Most incidents occurred when staff did not provide medication assistance in accordance with the established procedures for some reason. In some cases, they were distracted by unexpected events. The need to carry out tasks other than medication assistance in parallel created a “mental burden” for caregivers, leading to "impatience" and "assumptions," which in turn triggered medication errors. Lack of knowledge about the medications being administered was also a consideration, as was experience of medication assistance. Unfamiliarity with medication assistance due to lack of experience could lead to errors in the procedures, while over-familiarity with the work could lead to the omission of a part of the procedures.

### Team factor

In an environment where the work is complex, cooperation with other staff is considered necessary for smooth medication assistance, but it was sometimes difficult to get sufficient support. “Failures to communicate with other staff” might lead to impatience in performing procedures, which could lead to medication incidents.

### Work environment factor

In this study, all incidents had occurred in the cafeteria, a space designed for a large number of people to eat together. A situation had been created where many residents were present at the same time, and persons with similar names were in close proximity. The timing of starting to eat their meals varied. These situations constantly reminded the staff of “presence of other residents” and “other work besides medication assistance”. Moreover, “spatial arrangement of residents”, in that people with similar names were in close proximity to each other, could also lead to incidents.

It was pointed out that the work was hard and sometimes overwhelming, resulting in "not enough time”. Time availability for medication assistance varies depending on the number of persons who can assist. Staff felt that “lack of manpower” had resulted in medication incidents.

In addition, it was pointed out that simplification of procedures was routinely implemented due to the intense workload and that the nature of previous incidents was not sufficiently shared. In such an environment, the facility might show “little understanding of fostering a safety culture”.

## Discussion

This study focused on the medication incidents that occurred during medication assistance by staff who were not doctors or nurses, and clarified the factors contributing to medication incidents. These incidents occurred in situations where staff could not keep to the established procedures, and contributory factors were classified into 4 categories; individual resident, individual staff, team, work environment.

In this study, none of the incidents resulted in a serious outcome. In Japan, the scope of medication assistance by caregivers who are not doctors or nurses is very limited, and the care system of medication assistance by caregivers is designed to prevent incidents with serious outcomes. Therefore, this study extracted factors that contribute to incidents related to medication assistance by non-medical professionals in residential care facilities.

Factors contributing to medication errors in hospitals or long-term care facilities have already been reported [4–7]. Patient-related risk factors for medication errors included polypharmacy, increased age, number of diseases or comorbidities, female gender, low level of education, and so on [6]. These factors were not extracted in our study. It was also reported that nurse-related contributory factors to medication errors were “proper protocol not followed”, “lack of knowledge”, “inappropriate communication" [7]. These factors were extracted in our study. On the other hand, factors such as “presence of other residents” or “spatial arrangement of residents” identified in this study appear to be unique to medication assistance in residential care facilities in Japan. The reason may be that medication assistance by staff in residential care homes is generally conducted in a cafeteria where many residents eat together. Thus, the work environment of medication assistance may be more complicated than that at home or in a hospital. These results suggest that it may be useful to establish a system in which the meal times and medication assistance are separated. If all residents received medication assistance in their own rooms, this might reduce the risk of medication incidents.

There is a heavy workload in residential care homes, and it may also be important to reduce the workload from the viewpoint of risk management. However, in practice, it might be difficult to increase staffing levels. Furthermore, few care facilities in Japan have a resident pharmacist, and pharmacists may not be able to provide adequate support in residential care facilities in Japan [8–10]. The situation might be improved if pharmacists could monitor the medication status of the entire facility and review the timing of medication administration for each resident (so that, for example, the timing of medication could be staggered to reduce the workload at one time, such as at breakfast).

Vincent et al. reported 7 contributory factors for medical errors in clinical tasks: patient, task/technology, staff, work environment, organization/management, and institutional context [11]. Our survey identified 5 of these factors (the organization/management factor included in the work environment factor), but not task/technology, which is thought to be related to the design or procedure of medication assistance, or organization/management, or institutional context. These results suggest that from the caregivers’ perspective, the procedures for medication assistance may be appropriate, at least in the facilities included in this study.

There are a number of potential limitations to the present study. First, the subjects included in this study were limited to persons in care facilities operated by a single company. The facilities involved had two features in common: residents have meals in a cafeteria, and almost all residents receive medication assistance from staff. The procedures for medication assistance were common in all of the facilities. Second, all incidents covered in this study were incidents in which the wrong resident was given the medication. Other contributory factors might have been extracted if other incidents, such as wrong time of administration or omission of medication, had been involved. Third, only incidents recognized by staff in residential care facilities were included in the analysis. Fourth, data collection was conducted by pharmacy researchers and the company's business support staff, and data analysis was mainly conducted by a pharmacy researcher. These persons could potentially have influenced collection and interpretation of the data. Finally, in this study we did not use the method of coding by two independent persons. Instead, coding was conducted by the first author (KH), then the generated codes and themes were reviewed by another author (SH), and finalized by discussion and agreement between KH and SH.

This study is the first to evaluate caregivers’ perspectives about contributory factors to medication incidents associated with medication assistance for care home residents provided by staff who are not medical professionals. Our findings should be helpful for risk management relating to medication incidents. In particular, we suggest that separation of meal times and medication assistance, and professional review of the timing of administration of residents’ medication may be effective in reducing incidents. Future studies will be needed to examine whether such operational changes and professional interventions would actually reduce medication incidents.

## Supplementary Information

**Additional file 1.** 

## Data Availability

The raw verbatim interview data used in the current study are not publicly available because informed consent was not obtained for publication of participant data. Upon reasonable request, the anonymized analyzed data may be obtained from the corresponding author.
