# Supplementary material for: Analysis of contributory factors to incidents related to medication assistance for residents taking medicines in residential care homes for the elderly: a qualitative interview survey with care home staff
Source: BMC Geriatr. 2022 Apr 22;22:352. doi: 10.1186/s12877-022-03016-4 (PMC9027828; doi:10.1186/s12877-022-03016-4)
Supplement: Supplementary file 1 — Additional file 1. [file 12877_2022_3016_MOESM1_ESM.docx]

Supplementary Information

Appendix 1 Interview guide

This is an English translation of the Japanese language interview guide. The interview survey was conducted in Japanese.

**Basic information**

-Experience (years) of caregivers

-Care system or meals in the facility

-Features of the facility

-Burden of medication assistance

-Difficulties that staff have experienced

**Questions about how the incident occurred**

-When or how did the incident occur?

-When or how was the incident identified?

-Features of the resident involved

-Actions after the medication incident

-Impact of the medication incident

**Contributory factors and counter-measures**

-Contributory factors to the incident in caregiver’s opinion

-Counter-measures for the incident

-Factors including software, hardware, environment, and management

-Learning from the incident

Appendix 2 Procedures of medication assistance (medication after meals)

1. Check food intake of the resident, and whether the resident is ready for medication.

2. Find the resident’s medication on the medication cart, and check the match between resident’s name on the box and on the medicine envelope.

3. Place water and the medication box in the special tray for medication distribution.

4. Take the tray to the resident who will take the medication.

5. At the table, pick up the nameplate on the table and set it and the medicine envelope on the tray.

6. Check the match between the name on the medicine envelope and the resident’s name.

7. Read aloud the information on the medicine envelope (resident’s name/date/time of medication, etc.), and check it with the resident.

8. Encourage the resident to drink water and wet his/her mouth.

9. Give the resident the medication. According to the condition of the resident, hand over the medicine envelope, or open the medicine envelope and put it on the resident’s hand, or put the contents in a medicine cup and hand it over, etc.

10. Make sure the resident doesn’t spill anything.

11. Make sure there are no pills left in the medicine envelope.

12. Return the medication box to the medication cart.
